# Angiogenic factors and their soluble receptors predict organ dysfunction and mortality in post-cardiac arrest syndrome

**DOI:** 10.1186/cc11648

**Published:** 2012-09-29

**Authors:** Takeshi Wada, Subrina Jesmin, Satoshi Gando, Yuichiro Yanagida, Asumi Mizugaki, Sayeeda N Sultana, Sohel Zaedi, Hiroyuki Yokota

**Affiliations:** 1 Division of Acute and Critical Care Medicine, Department of Anesthesiology and Critical Care Medicine, Hokkaido University Graduate School of Medicine, N17W5, Kita-ku, Sapporo 060-8638, Japan; 2 Department of Emergency and Critical Care Medicine, Faculty of Medicine, University of Tsukuba, 1-1-1, Tennoudai, Tsukuba, Ibaraki 305-8575, Japan; 3 Health and Diseases Research Center for Rural Peoples (HDRCRP), 14/15, 1st floor, Probal Housing Ltd., Shekertak (Adjacent to Shekertak Road 1), Mohammadpur, Dhaka 1207. Bangladesh; 4 Department of Emergency and Critical Care Medicine, Nippon Medical School, 1-1-5 Sendagi Bunkyo-ku, Tokyo 113-8603, Japan

## Abstract

**Introduction:**

Post-cardiac arrest syndrome (PCAS) often leads to multiple organ dysfunction syndrome (MODS) with a poor prognosis. Endothelial and leukocyte activation after whole-body ischemia/reperfusion following resuscitation from cardiac arrest is a critical step in endothelial injury and related organ damage. Angiogenic factors, including vascular endothelial growth factor (VEGF) and angiopoietin (Ang), and their receptors play crucial roles in endothelial growth, survival signals, pathological angiogenesis and microvascular permeability. The aim of this study was to confirm the efficacy of angiogenic factors and their soluble receptors in predicting organ dysfunction and mortality in patients with PCAS.

**Methods:**

A total of 52 resuscitated patients were divided into two subgroups: 23 survivors and 29 non-survivors. The serum levels of VEGF, soluble VEGF receptor (sVEGFR)1, sVEGFR2, Ang1, Ang2 and soluble Tie2 (sTie2) were measured at the time of admission (Day 1) and on Day 3 and Day 5. The ratio of Ang2 to Ang1 (Ang2/Ang1) was also calculated. This study compared the levels of angiogenic factors and their soluble receptors between survivors and non-survivors, and evaluated the predictive value of these factors for organ dysfunction and 28-day mortality.

**Results:**

The non-survivors demonstrated more severe degrees of organ dysfunction and a higher prevalence of MODS. Non-survivors showed significant increases in the Ang2 levels and the Ang2/Ang1 ratios compared to survivors. A stepwise logistic regression analysis demonstrated that the Ang2 levels or the Ang2/Ang1 ratios on Day 1 independently predicted the 28-day mortality. The receiver operating characteristic curves of the Ang2 levels, and the Ang2/Ang1 ratios on Day 1 were good predictors of 28-day mortality. The Ang2 levels also independently predicted increases in the Sequential Organ Failure Assessment (SOFA) scores.

**Conclusions:**

We observed a marked imbalance between Ang1 and Ang2 in favor of Ang2 in PCAS patients, and the effect was more prominent in non-survivors. Angiogenic factors and their soluble receptors, particularly Ang2 and Ang2/Ang1, are considered to be valuable predictive biomarkers in the development of organ dysfunction and poor outcomes in PCAS patients.

## Introduction

There have been progressive improvements in the management of cardiac arrest, including modern cardiopulmonary resuscitation and emergency cardiovascular care. Nevertheless, the prognosis of successfully resuscitated patients remains poor, and life-threatening disturbances known as "post-resuscitation disease" or "post-cardiac arrest syndrome (PCAS)" can lead to multiple organ dysfunction syndrome (MODS) [1]. Endothelial and leukocyte activation after whole-body ischemia/reperfusion following resuscitation from cardiac arrest is a critical step in endothelial injury and related organ damage [2]. Prolonged ischemia results in severe tissue and organ damage, reperfusion-induced injury; defined as tissue damage directly related to revascularization; may be even more harmful [3,4].

Vascular endothelial growth factor (VEGF) plays crucial roles in angiogenesis and microvascular permeability [5]. VEGF signaling in endothelial cells releases cytokines and chemokines, and induces the expression of procoagulant and cell adhesion molecules. VEGF primarily binds to two transmembrane receptors, VEGF receptor (VEGFR)-1 and VEGFR2. VEGFR2 is selectively expressed in the endothelium and mainly mediates endothelial growth, survival signals and pathological angiogenesis. In contrast, VEGFR1 is present on both endothelial cells and monocytes, and VEGFR1-mediated signaling plays important roles by increasing the vascular permeability under pathological conditions, such as ischemia and inflammation.

The angiopoietin (Ang)-Tie2 ligand-receptor system is restricted to the regulation of the endothelium and is involved in multiple MODS-related pathways [6]. The Ang-Tie2 system not only regulates angiogenesis, but also controls endothelial inflammation, along with VEGF and its receptor system [7,8]. Ang1 stabilizes endothelial cells, inhibits vascular leakage, and suppresses inflammatory and coagulation-related gene expression through Tie2 activation [8-10]. Ang2 antagonizes the binding of Ang1 to Tie2. Therefore, Ang2 is thought to act as a proinflammatory mediator by increasing fluid leakage through the endothelial vasculature [11]. Several studies have demonstrated that the ratio of Ang1 to Ang2 better describes the state of activation of the endothelium, because Ang1 and Ang2 exhibit agonist-antagonist effects on the endothelium [12,13].

Many studies have demonstrated a relationship between the pathophysiology of sepsis and the activities of angiogenic factors, including VEGF, angiopoietins and corresponding receptors. We have observed a relationship between angiogenic factors, their receptors and disseminated intravascular coagulation (DIC) associated with sepsis [14]. In addition, we have demonstrated the presence of a pathophysiological relationship between angiogenic factors and their soluble receptors and organ dysfunction in patients with DIC associated with severe trauma [15]. However, no previous reports have documented data regarding angiogenic factors and their soluble receptors in patients with PCAS. The aim of this study were to test the hypothesis that angiogenic factors and their soluble receptors play pivotal roles in the development of organ dysfunction related to PCAS, thus leading to a poor outcome, and to confirm the efficacy of these factors as prognostic biomarkers of organ dysfunction and mortality in PCAS patients.

## Materials and methods

### Patients

This study was performed from May 2001 until April 2008. Approval for this study was obtained from the institutional review board, the Ethics Committee of Hokkaido University School of Medicine. Informed consent for this study was obtained from the patients' next of kin. Cardiac arrest was defined as the absence of a palpable pulse of the common carotid artery confirmed by an emergency medical service worker. Patients were excluded if they were younger than 18 years of age or had a terminal illness or history of trauma-induced arrest. Cardiopulmonary resuscitation was performed in accordance with the Guidelines 2000 for Cardiopulmonary Resuscitation and Emergency Cardiovascular Care [16]. A total of 52 patients resuscitated after out-of hospital cardiac arrest from May 2001 to April 2008 were enrolled in the study. The patients were subdivided into survivors and non-survivors according to their 28-day mortality. All patients were also divided into MODS and non-MODS groups. Fifteen healthy adult volunteers served as the control subjects.

### Definitions

The patients' severity of illness was evaluated according to the Acute Physiology and Chronic Health Evaluation (APACHE) II score determined after the first 24 hours of admission [17]. Organ dysfunction was assessed according to the Sequential Organ Failure Assessment (SOFA) score [18]. Multiple organ dysfunction syndrome (MODS) was defined as a SOFA score > 12 [18]. Overt DIC scores based on the International Society on Thrombosis and Haemostasis (ISTH) criteria were calculated [19]. A diagnosis based on the ISTH criteria was established when the total score was > 5. We defined the maximum score (max) as the highest score observed during the study period. The main outcome measure was 28-day mortality.

### Study protocol and measurement methods

Blood samples were collected using an arterial catheter within 24 hours of arrival at the emergency department (Day 1), and on Days 3 and 5. The blood samples were immediately placed into individual tubes and centrifuged at 3,000 rpm, for five minutes at 4°C. The serum and/or plasma were stored at -80°C until used for the assay.

The following variables were measured in duplicate: VEGF (Human VEGF, Quantikene; R&D Systems, Inc., Minneapolis, MN, USA); soluble VEGF receptor (sVEGFR)1 (Human sVEGF R1/Flt-1, Quantikene; R&D Systems, Inc.); sVEGFR2 (Human sVEGF R2/KDR/Flk-1, Quantikene; R&D Systems, Inc.); Ang1 (Human Angiopoietin-1, Quantikene; R&D Systems, Inc.); Ang2 (Human Angiopoietin-2, Quantikene; R&D Systems, Inc.); and soluble Tie2 receptor (sTie2) (Human Tie-2, Quantikene; R&D Systems, Inc.).

### Statistical analysis

The statistical analyses and calculations were performed with the SPSS 19.0 software package (SPSS, Inc., Chicago, IL, USA). Differences between groups were analyzed using a two-sided nonparametric Mann-Whitney U test, and categorical variables were compared using Pearson's chi-square test or Fisher's exact test when required. The Shapiro-Wilk test was used for statistical testing of normality. Logarithmic transformations were made for all variables when needed. A stepwise logistic regression analysis was used to assess the relationship between the 28-day mortality and age, gender, the APACHE II score ISTH DIC score on Day 1 and the levels of VEGF, sVEGFR1, sVEGFR2, Ang1, Ang2, Ang2/Ang1, sTie2, the logarithmic transformation form of Ang2 (Ang2(log10)), and Ang2/Ang1(log10) on Day 1. A multiple regression analysis was also performed to assess the relationship between the SOFA score max and the same variables. Variables found to be statistically significant at a 10% level in the univariate analysis were included in the multivariate model. Receiver operating characteristic (ROC) curves were constructed for the outcome (death), based on the levels of Ang2, the Ang2/Ang1 ratios on Day 1. The areas under the ROC curves (AUC) with standard error (SE) were examined using a significance test for AUC. The optimal cutoff value was determined using the Youden index. A *P*-value < 0.05 was considered to be statistically significant. All results are expressed as the means + SEM, unless otherwise stated.

## Results

### Patients' characteristics

The causes of cardiac arrest are shown in Table 1. There were no significant differences between the two groups. Table 2 presents the characteristics of the patients in the two groups. There was a significantly higher proportion of females among the non-survivors. A witnessed arrest, bystander cardio-pulmonary resuscitation (CPR) and the time intervals did not significantly differ between survivors and non-survivors. There were significant differences in the defibrillation attempt and the doses of adrenalin. The non-survivors demonstrated higher ISTH DIC score max, APACHE II scores, more severe degrees of organ dysfunction (SOFA score max) and a higher prevalence of MODS.

**Table 1:** The causes of cardiac arrest

	Surviviors (*n *= 23)	Non-survivors (*n *= 29)
Cardiovascular	11	8
Respiratory	4	6
Neurogenic	2	5
Asphyxia	6	9
Undetermined	0	1

**Table 2:** The baseline clinical characteristics of survivors and non-survivors of PCAS patients

	Survivors (*n *= 23)	Non-survivors (*n *= 29)	*P *value
Age (years)	62 ± 5	66 ± 4	0.423
Gender (male/female)	20/3	13/16	0.002
Witnessed arrest (yes/no)	12/11	14/15	0.780
Bystander CPR (yes/no)	7/16	10/19	0.757
Initial rhythm (Vf/Asystole/PEA/unknown)	6/8/7/1	1/19/7/2	0.059
Time interval (min)*			
1	6.5 ± 0.6	6.2 ± 0.4	0.874
2	13.9 ± 1.3	13.2 ± 1.5	0.483
3	33.4 ± 2.1	32.5 ± 1.5	0.919
4	35.3 ± 2.9	34.8 ± 2.3	0.846
Defibrillation (yes/no)	9/14	3/26	0.014
Adrenalin (mg)	1.4 ± 0.3	2.6 ± 0.4	0.044
APACHE II score	26.4 ± 1.4	34.3 ± 1.1	< 0.001
SOFA score max	5.1 ± 0.5	8.7 ± 0.7	0.001
MODS (yes/no)	1/22	9/20	0.015
ISTH DIC score max	1.7 ± 0.3	3.0 ± 0.3	0.006

*1, interval between the receipt of the emergency call and arrival of the vehicle; 2, interval between the arrival of the vehicle and the arrival at the ED; 3, interval between the receipt of the emergency call and the arrival at the ED; 4, total CPR time; APACHE, Acute Physiology and Chronic Health Evaluation; CPR, cardio-pulmonary resuscitation; DIC, disseminated intravascular coagulation; ISTH, International Society on Thrombosis and Haemostasis; max, maximum score; MODS, multiple organ dysfunction syndrome; PEA, pulseless electrical activity; SOFA, Sequential Organ Failure Assessment; Vf, ventricular fibrillation

### Serial changes in values of angiogenic factors and their soluble receptors

**Figure 1:**
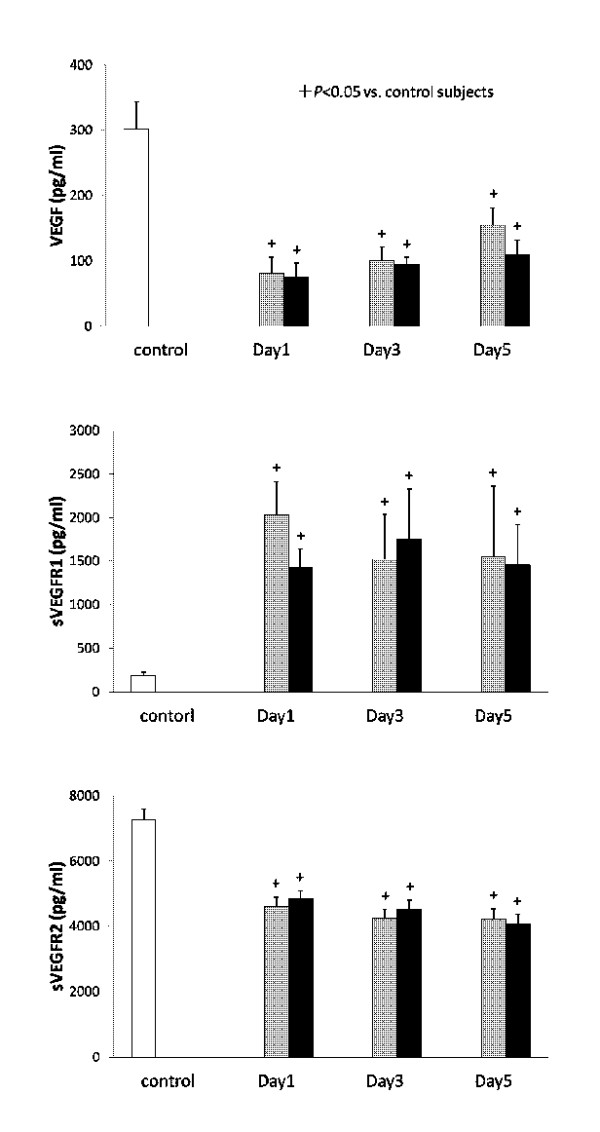
**The levels of VEGF, sVEGFR1 and sVEGFR2**. White bars, control subjects; gray bars, survivors; black bars, non-survivors.

**Figure 2:**
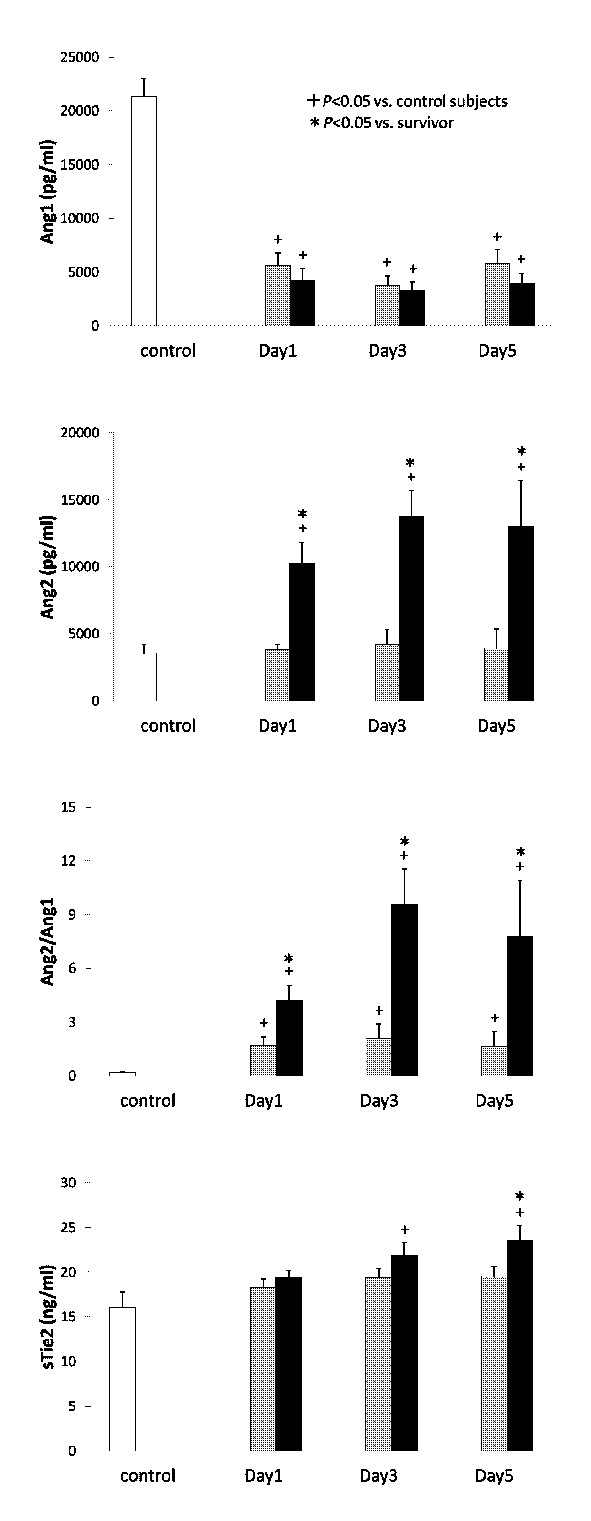
**The levels of Ang1, Ang2, sTies2 and the Ang2/Ang1 ratios**. White bars, control subjects; gray bars, survivors; black bars, non-survivors.

Serial changes in the circulating levels of VEGF, sVEGFR1 and sVEGFR2 are presented in Figure 1. The resuscitated patients showed significantly higher levels of sVEGFR1 and lower levels of VEGF and sVEGFR2 in comparison to the controls. The levels of VEGF, sVEGFR1 and sVEGFR2 did not show any significant differences between the survivors and the non-survivors during the entire study period. Figure 2 presents the levels of Ang1, Ang2, sTie2 and the Ang2/Ang1 ratio. Although the Ang1 levels in the resuscitated patients were significantly lower than those in the control subjects, no statistically significant differences were observed between the survivors and the non-survivors. On the contrary, the Ang2 levels in the non-survivors were significantly higher than those in the control subjects and the survivors throughout the study period. Therefore, the Ang2/Ang1 ratios significantly increased in the non-survivors. In addition, the sTie2 levels measured on Day 5 were higher in the non-survivors than in the survivors.

### Relationships between angiogenic factors, their soluble receptors and mortality

**Figure 3:**
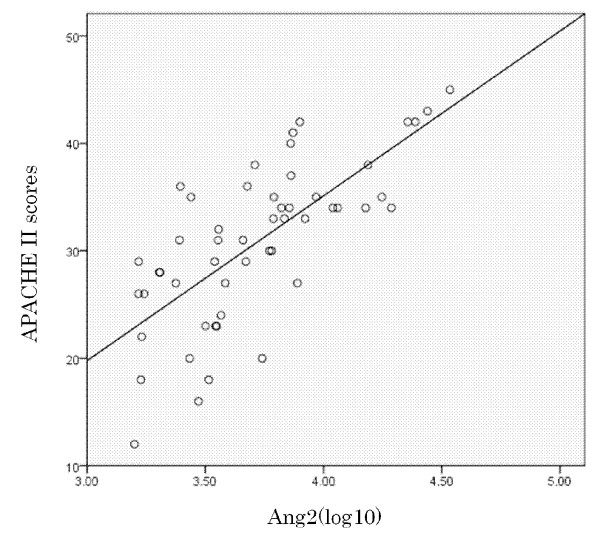
**Scatter plots showing the correlations between Ang2(log10) and the Acute Physiology and Chronic Health Evaluation (APACHE) II score in 52 resuscitated patients (survivors (*n *= 23), non-survivors (*n *= 29))**. *r*^2 ^= .521, *P *< .001.

**Figure 4:**
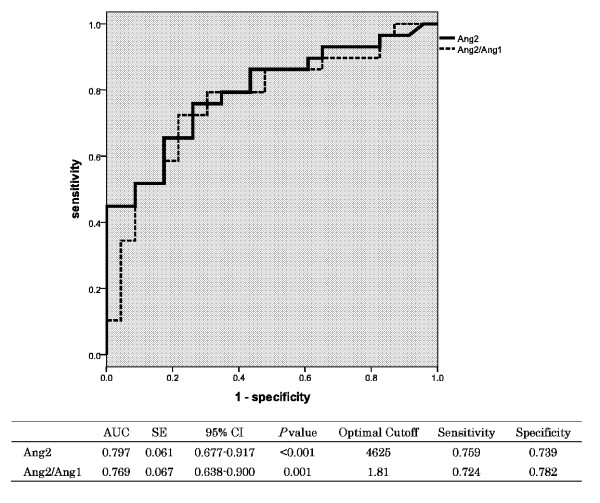
**The receiver operating characteristic (ROC) curve analysis for the outcome (death)**.

A linear regression analysis detected a strong association between Ang2(log10) and the APACHE II scores (*r*^2 ^= .521, *P *< .001; Figure 3). Table 3 shows that the Ang2 level or the Ang2/Ang1 ratio was identical as a strong, independent prognostic factor for 28-day mortality in this cohort of PCAS patients. APACHE II score and the ISTH overt DIC score on Day 1 also predicted 28-day mortality in PCAS patients. The ROC curves of the Ang2 levels and the Ang2/Ang1 on Day 1 used to predict 28-day mortality and the AUC (SE), 95% confidence interval (CI), optimal cutoff point value, sensitivity and specificity of the ROC curves are shown in Figure 4. Both values were found to be good predictors of 28-day mortality.

**Table 3:** The results of the univariate and multivariate logistic regression analysis for predicting 28-day mortality in PCAS patients

	Univariate			Multivariate		
**Variables**	**OR**	**95% CI**	***P *value**	**OR**	**95% CI**	***P *value**

Age (years)	1.010	0.983-1.038	0.455			
Gender (m/f)	8.205	1.989-33.847	0.004	21.5	2.769-166.4	0.003
ISTH DIC score	1.630	1.093-2.431	0.017	2.016	1.131-3.753	0.018
VEGF	1.000	0.995-1.004	0.862			
sVEGFR1	1.000	0.999-1.000	0.166			
sVEGFR2	1.000	1.000-1.001	0.507			
Ang1	1.000	1.000-1.000	0.386			
Ang2/Ang1^a^	1.401	1.028-1.910	0.033	1.381	1.044-1.827	0.024
Ang2/Ang1(log10)^a^	7.488	1.870-29.987	0.004	15.769	2.281-109.01	0.005
Ang2^ab^	1.000	1.000-1.001	0.007	1.000	1.000-1.001	0.018
Ang2(log10)^ab^	56.524	4.803-665.225	0.001	90.484	3.383-2420.48	0.007
sTie2	1.055	0.929-1.199	0.410			
APACHE II^ab^	1.223	1.085-1.378	0.001	1.206	1.044-1.394	0.011

Ang2(log10), *P *= 0.414; APACHE II, *P *= 0.151). CI, confidence interval; OR, odds ratio.

^a ^Different models were established, incorporating either the Ang2/Ang1, Ang2/Ang1(log10), Ang2, Ang2(log10), and APACHE II score, respectively. The models were tested separately.

^b ^ISTH DIC did not remain significant in these models, (Ang2, *P *= 0.363)

### Relationships between angiogenic factors, their soluble receptors and organ dysfunction

**Figure 5:**
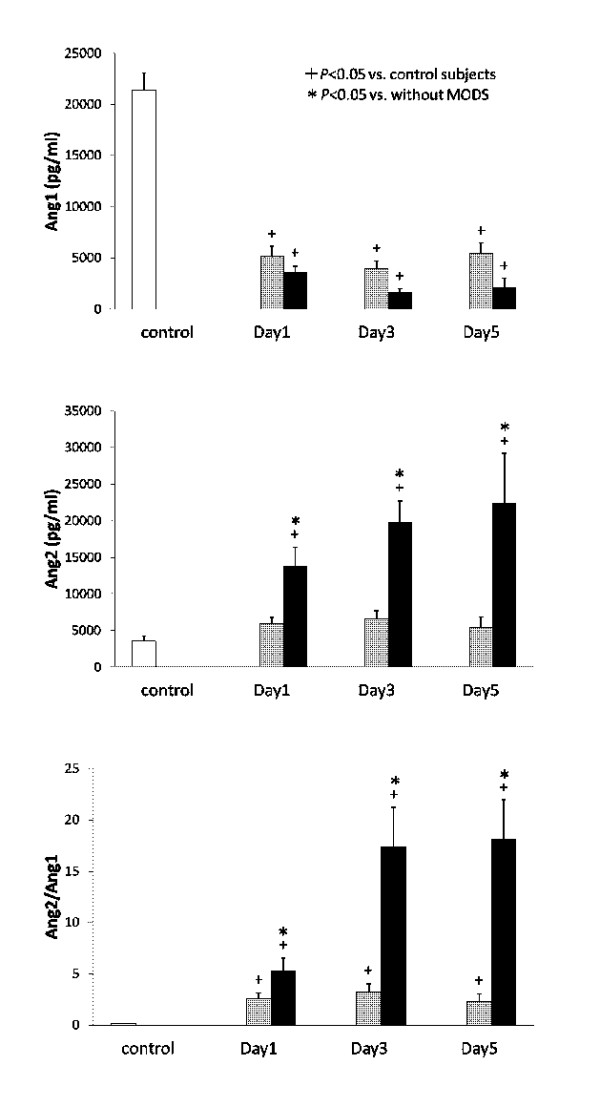
**The levels of Ang1, Ang2 and the Ang2/Ang1 ratios in the serum of PCAS patients**. White bars, control subjects; gray bars, non-MODS; black bars, MODS.

Figure 5 shows the serial changes in the circulating levels of Ang1, Ang2 and the Ang2/Ang1 ratios in the non-MODS and MODS groups. The Ang2 levels in the non-MODS group were identical to those in the control subjects; however, those in the MODS group were significantly higher than those in the control subjects and the non-MODS group during the study period. Therefore, the Ang2/Ang1 ratios significantly increased in the MODS group during the study period. A multiple linear regression analysis suggested that Ang2(log10) was an independent best predictor of changes in SOFA(log10) (Table 4).

**Table 4:** The results of a multiple regression analysis using the stepwise method for predicting the SOFA score max in PCAS patients

	B	SE	ß	*P *value	95% CI
SOFA(log10)					
Ang2(log10)	0.338	0.087	0 .489	< 0.001	0.163-0.513

*R*^2 ^= 0.224, ANOVA *P *< 0.001

ß, standard partial regression coefficient; ANOVA, analysis of variance. B, partial regression coefficient; CI, confidence interval; R^2^, coefficient of determinant; SE, standard error. Potential predictive variables included logarithmic transformation of age, APACHE II score, and levels of all angiogenic factors and their receptors on Day 1.

## Discussion

The present study found that Ang2 plays a pivotal role in the development of organ dysfunction associated PCAS, thus leading to a poor outcome. Moreover, the Ang2 level or the Ang2/Ang1 ratio can predict the development of organ dysfunction and mortality in patients with PCAS.

Lower VEGF levels are also associated with organ dysfunction and a poor outcome in patients with sepsis [20,21]. We have demonstrated that patients with DIC associated with severe trauma show lower levels of VEGF [15]. On the other hand, several studies have reported that the plasma VEGF levels in patients with septic shock are higher than those in patients without shock, and that the VEGF concentration at the time of admission correlates with the severity of disease [22,23]. In addition, sVEGFR1, which is generated by alternative splicing of VEGFR1 mRNA and functions as a decoy molecule, competing with VEGFR1 for binding to VEGF, is correlated with morbidity and mortality and is a potent marker of disease severity in septic or critically ill patients [15,23-25]. Meanwhile, previous reports have suggested that VEGFR1 is involved in the migration of monocytes/macrophages, and that elevation of sVEGFR1 leads to an anti-inflammatory state [24,26]. Therefore, the significance of the levels of VEGF and sVEGFR1 in patients with critical illnesses, such as severe sepsis, septic shock and severe trauma remains controversial [12,15,24]. The present study found that PCAS patients have lower levels of VEGF and higher levels of sVEGFR1 than control subjects; however, the levels of VEGF and sVEGFR1 are not significantly different between survivors and non-survivors. These results suggest that the VEGF/VEGFR signaling pathways may play minor roles in the pathophysiology of PCAS. Similarly, in this study, the sVEGFR2 levels were not significantly different between survivors and non-survivors in PCAS patients. sVEGFR2 may have regulatory consequences with respect to VEGF-mediated angiogenesis. However, its precise role has not yet been clarified [27]. These results also indicate that sVEGFR2 may not play a major role in PCAS.

PCAS is often compared to "sepsis-like syndrome", because it is characterized by high levels of circulating cytokines and adhesion molecules and the dysregulated leukocyte production of cytokines [3,4]. Previous studies have shown lower Ang1 and higher Ang2 levels to be associated with poorer outcomes in patients with sepsis or critical illness [9,20,28,29]. Ang1 exhibits anti-inflammatory properties and protects against vascular leakage, while Ang2 promotes inflammation and increases vascular permeability leading to the development of acute respiratory distress syndrome (ARDS) [8,9,30,31]. Moreover, positive relationships between Ang2 and inflammatory cytokines, such as tumor-necrosis factor (TNF)-alpha and interleukin (IL)-6 are observed in severe sepsis [28]. Therefore, an elevation in the Ang2 level and a decrease in the Ang1 level may reflect a pro-inflammatory state that is best summarized by the ratio of Ang1 to Ang2 [12,13]. The current study and previous studies suggest that reperfusion-induced endothelial injury is reflected in higher Ang2 levels, as well as imbalances of Ang1 and Ang2 (high Ang2/Ang1 ratios) that are associated with systemic inflammatory responses leading to organ dysfunction and death in PCAS patients. The administration of Ang1 protects the vasculature from leakage, thereby countering the potentially lethal actions of VEGF and inflammatory agents in animal experiments [32]. Correcting imbalances between Ang1 and Ang2 with administration of Ang1 or inhibition of Ang2 may, therefore, represent new therapeutic strategies for treating severe inflammatory illnesses such as PCAS.

PCAS is also similar to "sepsis-like syndrome" with respect to the coagulofibrinolytic abnormalities associated with cardiopulmonary resuscitation (CPR) and the return of spontaneous circulation. Whole-body ischemia and reperfusion- induced endothelial injury, contribute to thrombotic occlusion of the vessels following the activation of coagulation and the impairment of fibrinolysis [1,33-35]. These changes lead to DIC in patients resuscitated from cardiac arrest [34,35]. The current study demonstrates both the Ang2 levels and the ISTH overt DIC scores to be independent predictors of mortality in PCAS patients. We have demonstrated that Ang2 is one of the pathophysiological factors mediating organ dysfunction in patients with DIC associated with sepsis and severe trauma [14,15]. These results suggest that no matter the causes, Ang2 may play a crucial role in the development of organ dysfunction, thus leading to a poor outcome. In addition, the results of these studies support our hypothesis that all insults (trauma/surgery, infection and ischemia/reperfusion) may bring out similar nonspecific body responses, such as inflammation, neuroendocrine discharge, coagulation and fibrinolysis to maintain body homeostasis [36].

The current study has several limitations. The present data are not consecutive; however, we believe that there was no bias in the enrollment because we included all patients whose data were collected by the data collector in the present study. Table 2 shows that females had significantly higher mortality. This result is probably due to a type I error related to the small number of patients. The causes of cardiac arrest in the study are diverse, including acute coronary syndrome, asphyxia, subarachnoid hemorrhage and so on. However, we believe that the injuries caused by hypoxia/ischemia and subsequent reperfusion overwhelm any injuries associated with the specific cause of cardiac arrest. Introduction of routine therapeutic hypothermia, which may interfere with coagulation, was not performed because this study was completed before the publication of studies showing the benefits of therapeutic hypothermia in comatose survivors [37].

## Conclusions

In the present study, non-survivors with PCAS showed significant increases in the Ang2 levels and Ang2/Ang1 ratios in comparison to survivors throughout the entire study period. The Ang2 level or the Ang2/Ang1 ratio and the ISTH overt DIC scores on Day 1 were found to be strong predictors of 28-day mortality in PCAS patients. Ang2 also independently predicted increases in the SOFA scores. These results suggest that Ang2 or Ang2/Ang1 may be an informative predictor of the development of organ dysfunction and mortality in patients with PCAS. Additionally, angiogenic factors, in particular Ang2, may play important roles in the development of organ dysfunction, leading to death in PCAS patients. Correcting imbalances between Ang1 and Ang2 with the administration of Ang1 or the inhibition of Ang2 may, therefore, represent new therapeutic strategies for treating severe inflammatory illnesses, such as PCAS.

## Key messages

• The VEGF/VEGFR signaling pathways may play a minor role in the pathophysiology of PCAS.

• The Ang2 level, the Ang2/Ang1 ratio and the ISTH DIC score can predict 28-day mortality in PCAS patients.

• Ang2 is also an independent predictor of increasing SOFA scores.

• Angiogenic factors, in particular Ang2, may play important roles in the development of organ dysfunction, leading to death in PCAS patients.

## Abbreviations

Ang: angiopoietin; APACHE: Acute Physiology and Chronic Health Evaluation; ARDS: acute respiratory distress syndrome; AUC: area under the curves; CI: confidence interval; CPR: cardio-pulmonary resuscitation; DIC: disseminated intravascular coagulation; IL: interleukin; ISTH: The International Society on Thrombosis and Haemostasis; MODS: multiple organ dysfunction syndrome; OR: odds ratio; PCAS: post-cardiac arrest syndrome; ROC: receiver-operating characteristics; SE: standard error; SOFA: Sequential Organ Failure Assessment; TNF: tumor necrosis factor; VEGF: vascular endothelial growth factor; VEGFR: vascular endothelial growth factor receptor.

## Competing interests

The authors declare that they have no competing interests.

## Authors' contributions

TW analyzed the results, drew the diagrams and wrote the manuscript. SJ proposed the initial idea, established the immunoassays, performed and supervised the experiments, and reviewed the manuscript. SG proposed the initial idea, designed and supervised the research, identified patients, collected samples, provided clinical data and reviewed the manuscript. AM provided clinical data and reviewed the manuscript. SNS and SZ established the experiments. YY and HY reviewed the manuscript. All authors read and approved the final manuscript.
